# The diagnostic accuracy of AI-assisted diabetic retinopathy screening in primary care: a prospective validation study

**DOI:** 10.1080/02813432.2026.2683982

**Published:** 2026-06-12

**Authors:** Malene Krogh, Marie Ørskov, Thomas Lohne Nørgaard, Marie Germund Nielsen, Martin Bach Jensen, Morten Sig Ager Jensen, Henrik Vorum, Jette Kolding Kristensen

**Affiliations:** ^a^ Center for General Practice, Aalborg University, Aalborg, Denmark; ^b^ Department of Clinical Medicine, Faculty of Health, Aalborg University, Aalborg, Denmark; ^c^ Department of Ophthalmology, Aalborg University Hospital, Aalborg, Denmark; ^d^ The Clinical Nursing Research Unit, Aalborg University Hospital, Aalborg, Denmark; ^e^ Department of Health Science and Technology, Aalborg University, Aalborg, Denmark

**Keywords:** Artificial intelligence, diabetic retinopathy, type 2 diabetes, general practice, diagnostic accuracy

## Abstract

**Objectives:**

This study investigated the diagnostic accuracy of AI-assisted diabetic retinopathy screening in primary care, using ophthalmologist-led screening as the reference standard.

**Methods:**

Patients with type 2 diabetes attending routine appointments at 10 primary care clinics underwent AI-assisted screening, followed by re-screening at an ophthalmology clinic. The quality of fundus images captured in primary care was independently assessed, and diagnostic accuracy was evaluated by comparing AI-assisted results with ophthalmologist results, including sensitivity, specificity, PPV, NPV, and AUC. Two analyses were conducted: one including all images and one excluding those of poor quality.

**Results:**

Among 183 patients (336 images), 18.6% of images were classified as poor quality. When all images were included, the AI-assisted screening achieved a sensitivity of 73.7%, specificity of 90.2%, PPV of 31.1%, NPV of 98.3%, and AUC of 0.82. Excluding poor-quality images improved sensitivity to 80.0%, NPV to 98.7%, and AUC to 0.84. Additional ocular findings unrelated to diabetic retinopathy were observed in 96 patients, including confirmed or non-specific signs of glaucoma, cataract, age-macular degeneration, benign nevus and reduced visual acuity.

**Conclusion:**

AI-assisted screening in primary care shows potential for clinical application, but further validation in larger populations and improvements in image quality are needed before clinical implementation.

## 1. Introduction

Diabetic retinopathy (DR) is a serious microvascular complication of diabetes that can lead to irreversible vision loss if not detected and treated in time [1,2]. Regular diabetic retinopathy screening (DRS) can reduce the risk of blindness [3,4], yet participation rates remain suboptimal worldwide [5]. Socioeconomic factors, lack of awareness, and non-compliance are key factors affecting participation rates [6], as well as long travel distances and extended wait list at ophthalmology practices [7,8].

To address the challenge of suboptimal participation, the World Health Organization Europe has highlighted the potential role of artificial intelligence (AI) in optimizing DRS programs [6]. AI can automate grading processes in DRS that are typically carried out solely by retinal specialists [9], which has opened the opportunity to reconsider where and by whom DRS is delivered.

In Denmark, the main type 2 diabetes (T2D) treatment occurs in primary care, while patients are required to attend DRS separately at ophthalmologist practice [10]. Integrating DRS into primary care could consolidate diabetes care and potentially make it easier for patients to attend screening. This model has been highlighted by both T2D patients and primary care professionals as a means to enhance accessibility, improve convenience, and increase participation in DRS [8,11–14]. In such a setup, primary care could identify patients with signs of DR who need follow-up by an ophthalmologist, while those without DR could continue screening in primary care. Given the low prevalence of DR among T2D patients in Denmark [10], this would help ensure that only those who truly need specialist assessment are seen, sparing many an additional visit in their diabetes care pathway. However, to realize this potential in practice, key factors must be addressed, including workflow integration, staff training, acceptance among staff and patients, and, importantly, the validity of AI-assisted DRS results.

Retrospective validation studies have demonstrated that AI exhibits high diagnostic accuracy in detecting DR; meaning equally good or even better than human retinal graders [15–17]. Retrospective studies often rely on high-quality images captured by experienced ophthalmic photographers and wide-field cameras, which cannot be directly transferable to a DRS setup in primary care. Further, the importance of evaluating AI-assisted DRS through prospective studies within the intended setting is crucial, as its performance can decrease when applied to real-world data that differ from those used during algorithm training [18–20].

An increasing number of prospective studies have examined the validity of AI-assisted DRS within primary care settings [20–28], with various types of AI software employed; while some utilize custom-developed AI models fine-tuned in real-world settings, others rely on commercially available AI software. A common feature across all studies is the use of non-mydriatic cameras with a standard-field (45–50 degrees). High accuracy has been reported, typically with a sensitivity of ≥87% and specificity of ≥84% [20,27,29]. However, issues related to poor image quality have also been observed in varying degrees [20,21,24,25,28], attributed to factors such as dirty lenses, small pupils, cataract, or other retinal diseases. In several cases [20,21,24,25], the AI software assesses whether images are ungradable due to poor quality, allowing staff to retake images if necessary. Unfortunately, insufficient images of lower quality are not always identified by AI analysis, consequently leading to false results (false positives or false negatives) [21,24,25]. This poses a challenge. If primary care staff, rather than specialists, are managing DRS, the reliability of its results is crucial for successful implementation. Especially considering that patients with negative results must be able to leave the DRS without the risk of a false negative.

As introduced, there may be advantages of implementing DRS in primary care from stakeholders’ perspectives [8,11–14], and accuracy of the algorithms shows promising results, however challenges and questions remain. An aspect not previously addressed is how screening outcomes differ when carried out by primary care staff using standard-field cameras without mydriasis compared to traditional ophthalmologist-led screenings, including what the ophthalmologist examines and reports. Understanding the differences can help shape decisions about the future role of AI-assisted DRS in primary care.

This study therefore aims to investigate the diagnostic accuracy of AI-assisted DRS compared to traditional ophthalmologist-led screening. In addition, retinal image quality in primary care is assessed, and additional findings identified during ophthalmologist-led screening are reported. This study builds upon existing efforts to understand and improve AI-assisted DRS in primary care, with the goal of increasing attendance for DRS.

## 2. Method

### 2.1. Study design and setting

This study was designed as a prospective diagnostic accuracy study conducted in a real-world primary care setting. The study reflects an early-stage evaluation of AI-assisted screening in a Danish context, with pragmatic choices in study design to fit clinical workflow. Screenings were conducted at two clinical sites: (1) across 10 general practices in the North Denmark Region, where patients were recruited and underwent initial AI-assisted screening, and (2) at an ophthalmologist practice in the same region, where patients underwent a reference examination. The study was conducted from March 2022 to June 2023 and is reported in accordance with the STARD 2015 guidelines for reporting diagnostic accuracy studies.

### 2.2. Study population

The 10 primary care practices were responsible for recruiting patients for participation in AI-assisted DRS as part of routine diabetes care. Patients were eligible if they had a diagnosis of T2D, were aged 18–70 years, were able to communicate in Danish, were not blind, and were able to attend a follow-up DRS at the ophthalmologist practice.

Patients were enrolled using convenience sampling. Potential participants were identified either through T2D patient lists or in connection with scheduled diabetes consultations. Recruitment was conducted by telephone or during routine primary care visits, where eligible patients were invited to participate. As this was an early-stage evaluation, no formal sample size calculation was performed. Instead, emphasis was placed on including a heterogeneous group of general practices. Each participating practice was instructed to recruit as many patients as feasible during a one-month intervention period. Written informed consent was obtained from all participating practices and patients. The study was approved by the Medical Research Ethics Committees (MERC) under the Danish Ministry of Health (Approval No: 2200781, February 25, 2022). Further details regarding study population and recruitment procedures have previously been described [30].

### 2.3. Camera and AI-analysis software

AI-assisted DRS was performed using a non-mydriatic fundus camera (FundusScope, Rodenstock, Germany) [31] with a 45° field of view. The camera is equipped with a 12-megapixel sensor and LED flash, enabling acquisition of high-resolution retinal images (4096 × 3072 pixels). Automated pupil zoom, image capture, and flash functions are integrated into the system. A 10.1-inch tablet mounted on the camera served as the operational interface and provided internet access, allowing direct transfer of images to the analysis software. Retinal images were automatically uploaded for analysis immediately after acquisition. AI-based image analysis was performed using the RetinaLyze system (RetinaLyze A/S, Hørsholm, Denmark)[32]. The software applies support vector machine learning to identify the presence or absence of visible retinal changes consistent with DR, providing an automated screening classification rather than a clinical diagnosis. RetinaLyze has been commercially available since 2013, and its performance has been evaluated in a limited number of studies [32–34]. The technical principles of the software have previously been described in detail[35]. Briefly, the software detects red retinal lesions, which are highlighted with black circular markers. The total number of detected lesions determines the screening result, presented using a color-coded output: green (no DR changes), yellow (few changes), red (several changes, >3), or grey (insufficient image quality). Image analysis is completed within approximately 15 s. The software does not detect other DR features such as hard exudates, cotton wool spots, or neovascularization. The RetinaLyze software was provided free of charge for the purpose of this study.

### 2.4. Screening staff and training

Within each participating practice, one or more members of the primary care staff involved in diabetes management were designated to perform the AI-assisted DRS. The fundus camera was installed on-site, and staff received training covering both theoretical and practical aspects of DRS.

The training program, previously described in detail [36], consisted of instructional video material combined with supervised hands-on practice. Training sessions were conducted in the designated screening room shortly before the first patient appointment. The content included operation of the camera, evaluation of image quality, transfer of images to the AI software, communication of screening results to patients, and management of common technical or procedural challenges.

### 2.5. Patient screening timeline

**Figure 1.**
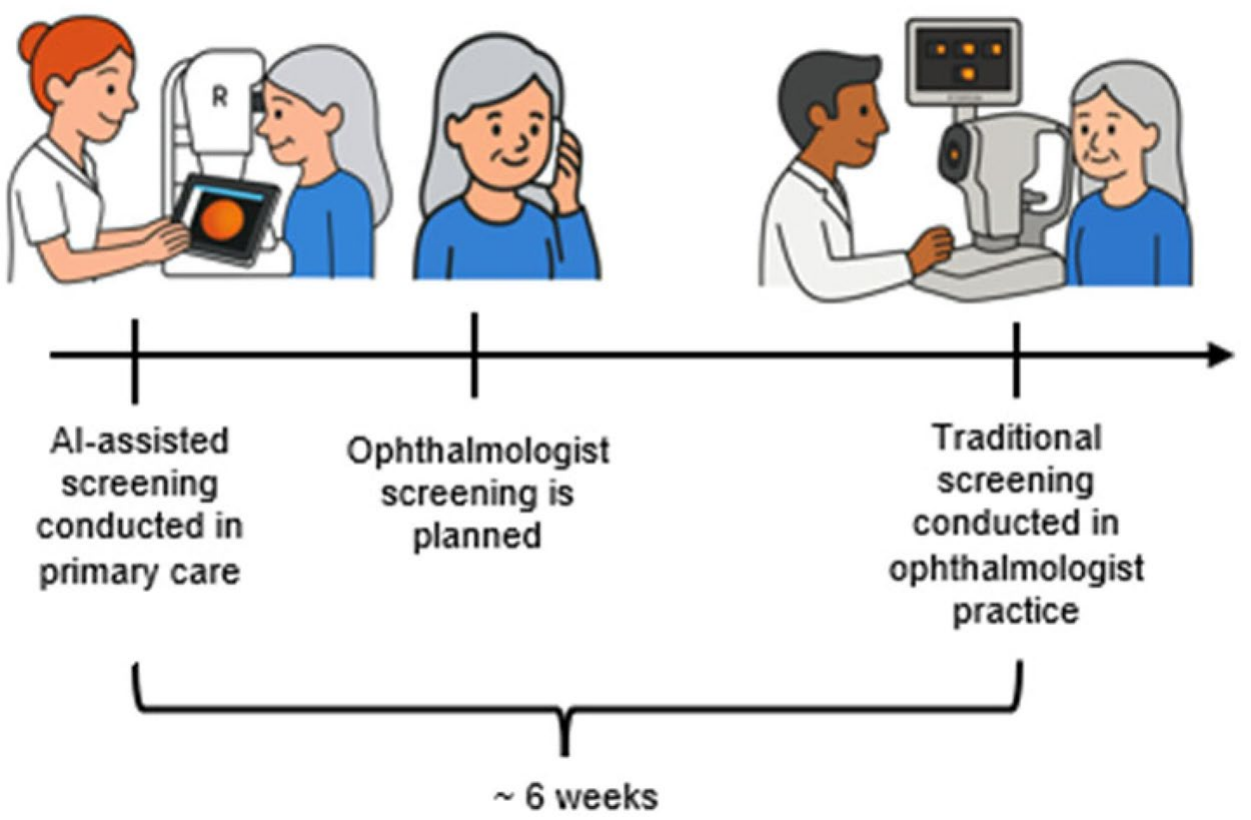
Patient screening timeline. Patients first undergo AI-assisted DRS in primary care, are then contacted by phone to schedule the next screening and finally attend a follow-up DRS at the ophthalmology clinic.

Patients were required to undergo two DRS in this study: one performed in primary care by their usual diabetes healthcare professional using AI analysis, and a second at the ophthalmologist practice (see Figure 1).

### 2.6. AI-assisted screening in primary care

Primary care staff screened patients without mydriasis during diabetes consultations. Staff were to follow these steps:

1. Darken the screening room as much as possible (turn off lights, close curtains).
2. Ensure correct patient positioning at the camera.
3. Capture fundus images of the patient’s eyes, with a wait time of at least 20 seconds between images to minimize interference from the flash and reduce pupillary reflex for the second photo.
4. Evaluate image quality manually.
5. Perform the analysis using the AI software. Retake photos if images are identified as insufficient.
6. Communicate the results to the patient.

After completion, screening staff recorded patient information, diabetes duration, date of latest DRS, HbA1c, and blood pressure on a tablet (Huawei MediaPad T3 10). The last two measurements were either newly obtained during the consultation or the most recent available for the patient.

### 2.7. Planning screening at ophthalmologist practice

As part of the screening in primary care, patients completed a questionnaire, which included providing their contact information. This information was provided to the ophthalmologist practice staff, who contacted patients by phone within 14 days after their initial screening to plan an appropriate date for the ophthalmologist screening. Multiple date and time options were presented for the patient. As a result, the interval between the two screenings varied slightly, with the follow-up screening generally conducted on average 6 weeks (SD 3 weeks) after the primary care screening. All patients received reimbursement for their transportation to the ophthalmologist practice.

### 2.8. Traditional screening at ophthalmologist practice and human grading procedure

Traditional DRS was carried out at ophthalmologist practices by staff specialized in retinal imaging. Each patient was allocated a 20-minute appointment, during which standard Danish screening procedures for patients with diabetes were followed. This included:

1. Measure visual acuity (Topcon CC-100 series)
2. Measure intraocular pressure and assess refraction (Topcon TRK-2P)
3. Provide patients with mydriasis eye drops (Mydriaticum Stulln, 1%).
4. Conduct fundus photos (Topcon 3D OCT – 1 Maestro2), with two images per eye (macula- and disc-centered images).

Retinal images were reviewed within a few days by a specialized retinal ophthalmologist, and findings were documented in a medical report. Diabetic retinopathy severity was graded according to the International Clinical Diabetic Retinopathy (ICDR) Severity Scale [37], which constitutes standard clinical practice in Denmark [38]. Any additional retinal findings were recorded and patients requiring further evaluation were contacted by telephone and advised to attend follow-up consultations or to contact their usual ophthalmologist.

For the purposes of this study, all retinal images obtained at the ophthalmology practices were independently graded by a second retinal ophthalmologist according to the ICDR Severity Scale [37]. Both ophthalmologists were blinded to each other’s assessments and to the results of the AI-based screening.

### 2.9. Quality check of images conducted in primary care

The quality of all images obtained in primary care was assessed by two specialized quality controllers, both of whom have extensive experience in evaluating retinal image quality. The image quality was evaluated using a scale from 1 to 3. A score of 1 indicated images that could be graded, with perfect quality and no defects that hindered the ability to grade the image. A score of 2 indicated images that could be graded but had minor flaws. A score of 3 indicated images that could not be graded due to major flaws, such as blurred images, significant artifacts, and other image quality issues.

### 2.10. Statistical analysis

Patient baseline characteristics were presented as means with standard deviations. Patients who did not attend their follow-up screening were excluded. Patients with missing values were retained in the analysis, and the available data were used. If only one retinal image was available from primary care, it was included and compared with the same eye from the reference screening, while the reference image from the missing eye image was excluded.

For image quality assessment, images scored as 1 or 2 were grouped ‘gradable’ while those scored as 3 were categorized as ‘ungradable’. In cases of disagreement of gradability between the quality graders, the respective images were categorized as ‘ungradable quality’. The percentages of ‘ungradable quality’ images were calculated.

Two diagnostic accuracy analyses were conducted: Analysis 1 which included all available images to reflect real-world conditions, and Analysis 2, which excluded images deemed to be ‘ungradable quality’ by the quality controllers to simulate future screening scenarios where low-quality images filtered out. Both analyses included sensitivity, to reflect the proportion of true positives correctly identified, and specificity, to reflect the proportion of true negatives [39]. Positive and negative predictive values (PPV and NPV) were used to assess the likelihood that a positive or negative test result corresponds to the actual disease status [39]. The area under the curve (AUC), derived from the receiver operating characteristic (ROC) curve, summarizes the relationship between sensitivity and specificity across all possible thresholds and provides a measure of the test’s overall accuracy. All analyses were reported with 95% confidence intervals. For comparison, screening results from both settings were dichotomized. For human graders, an ICDR score of 0 was categorized as ‘No DR present’, whereas scores from 1 to 4 were classified as ‘DR present’. For the AI software, a green result (‘no alterations’) was defined as ‘No DR present’, while yellow and red results (‘few’ or ‘several alterations’) were interpreted as ‘DR present’. Images for which the two human graders disagreed on the binary classification (No DR vs. DR) were excluded from the analysis.

Intergrader agreement was assessed using both percentages and Cohen’s Kappa for the quality controllers’ binary results and for the human graders’ results on both the ICDR and binary classification outcomes.

A descriptive analysis of the ophthalmologist’s additional findings was conducted, and the frequency of patients with additional findings was calculated. All findings were grouped into broader categories (Dry age-related macular degeneration (AMD), cataract, and glaucoma), covering cases ranging from mild to severe. Non-specific findings that do not represent a definitive diagnosis of AMD and Glaucoma but may indicate early signs were grouped into ‘Non-specific age-related macula findings’ and ‘Non-specific optic nerve head and pressure findings’. A ‘Reduced visual acuity’ category was included and contains only patients for whom reduced vision was noted as clinically relevant by the ophthalmologist, for example in relation to other non-specific findings that could indicate early signs of cataract.

## 3. Results

**Figure 2.**
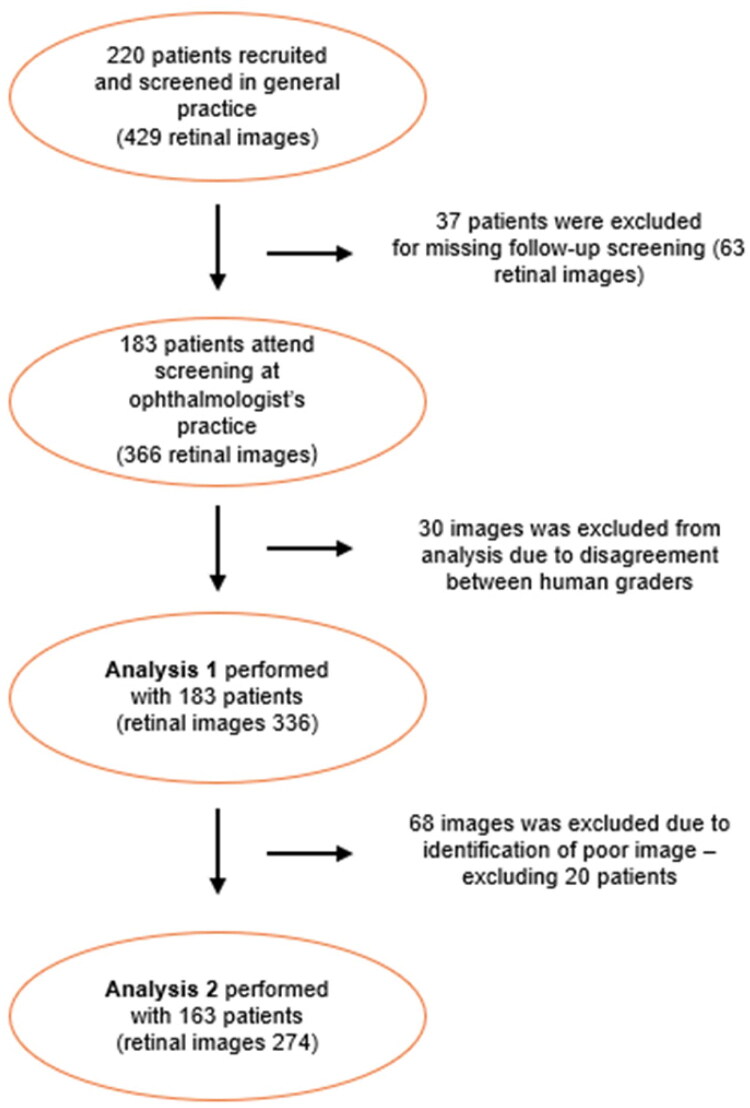
Flow of patients and retinal images in analysis. The figure shows how patients and images were included or excluded at different stages of the study. Exclusions occur at both patient level (missed follow-up screening) and image level (disagreement between human graders or poor image quality).

A total of 220 patients with T2D participated in the AI-assisted DRS in primary care, of whom 183 also completed the follow-up DRS conducted in ophthalmology practice. The remaining 37 patients could not be reached by phone, declined the follow-up screening, or did not attend their scheduled appointment. In total, 336 images were included in Analysis 1, comprising all available images from 183 patients. For Analysis 2, 274 images were retained after the exclusion of 62 images due to poor quality (excluding 20 patients as no images were to be analyzed). Patient flow and analysis steps are shown in Figure 2, and patient characteristics are presented in Table 1.

**Table 1.** Patient characteristics.

Variables	Summary	Missing / N (Pct)
n (%)	183 (100.0)	0 / 183 (0.0)
Gender, n (%)		
Male	108 (57.4)	
Female	78 (42.6)	0 / 183 (0.0)
Age, mean (SD)	59.3 (8.2)	0 / 183 (0.0)
T2D Duration, years (SD)	7.6 (5.7)	3 / 183 (1.6)
HbA1c, mmol/mol (SD)	57.8 (12.8)	3 / 183 (1.6)
Blood pressure, mmHg (SD)		
Systolic	128.6 (12.7)	
Diastolic	77.0 (10.5)	2 / 183 (1.1)
Latest DRS, n (%)		
<12 months	107 (58.5)	
>12 - <24 months	24 (13.1)	
>24 months	9 (4.9)	
None	17 (9.3)	26 / 183 (14.2)

T2D: Type 2 Diabetes, HbA1c: Hemoglobin A1c, DRS: Diabetic Retinopathy Screening.

### 3.1. Image quality

No images from general practice were identified by the AI software as being of poor image quality. In contrast, 68 images (18.6%) were assessed by the quality controllers as ungradable due to poor image quality. Mean age of patients whose images were assessed as ungradable was 62.7 ± 7.0 years, with the overall mean age in the sample was 59.3 ± 8.2 years (Table 1).

### 3.2. Diagnostic accuracy

A detailed overview of the cross-tabulation for both Analysis 1 and Analysis 2 is presented in Table 2. In Analysis 1, the five identified cases of DR included two cases of mild non-proliferative DR and three cases of moderate non-proliferative DR. In Analysis 2, among the three individuals identified with DR, one had mild non-proliferative DR and two had moderate non-proliferative DR. Table 3 presents the diagnostic accuracy metrics of both analyses side by side, for direct comparison.

**Table 2.** Cross-tabulation of DR classifications by human graders and AI software.

Analysis 1				Analysis 2			
	Human graders				Human graders		
AI software	No DR	DR	Total	AI software	No DR	DR	Total
**No DR**	286	5	291	**No DR**	230	3	233
**DR**	31	14	45	**DR**	29	12	41
**Total**	317	19	336	**Total**	259	15	274

Analysis 1 includes all available images; Analysis 2 excludes images identified as of poor quality. DR: diabetic retinopathy.

**Table 3.** Diagnostic accuracy for analysis 1 and analysis 2.

	Analysis 1	Analysis 2
Sensitivity	73.7% (95% CI: 51.2%–88.2%)	80.0% (95% CI: 51.9%-95.7%)
Specificity	90.2% (95% CI: 86.5%–93.0%)	89.0% (95% CI: 84.3%-92.3%)
PPV	31.1% (95% CI: 19.5%–45.7%)	29.3% (95% CI: 16.1%-45.5%)
NPV	98.3% (95% CI: 96.0%–99.3%)	98.7% (95% CI: 96.3%-99.7%)
AUC	0.82 (0.71–0.92)	0.84 (0.74–0.95)

Diagnostic performance measures of AI-assisted DRS compared with human grading. Sensitivity, specificity, positive predictive value (PPV), negative predictive value (NPV), and area under the curve (AUC) are presented with 95% confidence intervals. Adapted from the author’s PhD thesis[30].

### 3.3. Intergrader agreement between quality controllers and human graders

Intergrader agreement between the two quality controllers was 89.2%, with a Cohen’s kappa of 0.53. The intergrader agreement between the two human graders who assessed images using the ICDR scale was 92.8% with a Cohen’s kappa of 0.52. After data were consolidated into binary classification of DR or No DR, intergrader agreement was 94.8% and Cohen’s kappa was 0.64. The kappa values indicate moderate interrater agreement.

### 3.4. Additional findings at the ophthalmologist-led screening

Eighty-seven patients did not have any additional findings during their ophthalmologist-led screening, whereas 96 patients (52.2%) exhibited one or more additional findings. In total, 108 additional findings were observed, providing an average of 1.1 findings pr. patient. The number and percentage of patients with each additional finding, including the mean age (SD) for each category, are presented in Table 4.

**Table 4.** Additional findings from ophthalmologist report.

Additional finding	Number of patients	% of total patients (*n* = 183)	Mean age (SD)
Non-specific optic nerve head and pressure findings	45	24.6 %	61.2 (7.2)
Cataract	16	8.7 %	65.4 (4.4)
Non-specific age-related macular findings	14	7.7 %	56.4 (11.4)
Benign Nevus	12	6.6 %	61.0 (7.7)
Dry age-related macular degeneration	11	6.0 %	61.1 (6.9)
Glaucoma	5	2.7 %	61.8 (6.4)
Reduced visual acuity	5	2.7 %	63.8 (6.6)

Note: Patients may appear in more than one category.

## 4. Discussion

In this study, we investigated the diagnostic accuracy of performing AI-assisted DRS in primary care, thereby gaining insights into its performance under real-world conditions. Two separate analyses were conducted: one to provide a realistic representation of current screening conditions in primary care, and another to illustrate how screening performance could improve if poor-quality images were excluded at the time of acquisition. In both cases, patients without DR alterations were effectively identified, however the AI software reported a high portion of false positives.

When compared with other prospective studies conducted in primary care settings, which report sensitivities of ≥87% and specificities of ≥84% [20,27,29], our findings are broadly comparable. However, it is difficult to determine whether this level of diagnostic performance is acceptable for clinical implementation. To our knowledge, there is no universally accepted threshold for sensitivity and specificity in AI-assisted DRS. In Danish screening programs, tests are expected to be valid and clinically acceptable; however, what constitutes ‘acceptable’ performance depends on context and disease prevalence [40]. The observed results should therefore be interpreted in a screening context, where avoiding false-negatives is essential, as even small numbers may have clinical consequences. The aim in primary care is to detect DR while safely ruling out disease in patients without findings, making the balance between false negative and false positive critical. In this context, the high NPV (>98%) observed is particularly relevant, as it reflects the probability that a patient truly does not have DR following a negative test result[39]. However, this estimate should be interpreted considering the low disease prevalence in the study population. Further evaluation in larger and more diverse populations is required to assess its robustness and clinical utility before this screening approach can be considered a safe rule-out strategy for DR in primary care.

In this study, only a few false-negative results were observed. Nevertheless, false-negative results remain particularly concerning, as they may lead to patients being reassured and sent home despite having undetected DR. This can lead to clinical consequences for the patients if treatment is delayed, as it increase the risk of vision impairment if the condition remains undetected. In a primary care setting, it may therefore be preferable to accept some false positives rather than to miss true cases. The low PPV observed in our study indicates that some patients were incorrectly identified as having DR. This is not unexpected given the low disease prevalence, both in our sample and in Danish primary care in general [10]. In contrast, within Danish hospital settings where the prevalence of DR is higher[10], a correspondingly higher PPV can be expected, as demonstrated by Nissen et al. who used the same AI software, though with a slightly different study design and population, and observed a PPV of 73.6% and an NPV of 92.1% [33].

Across studies, AI models have failed to identify images of poor quality. Zhang et al. found that an AI model failed to flagg 15.8% of poor-quality images [21], while others noted that AI models exclude images deemed acceptable by human graders[28]. In this study, no images were flagged by the AI software as having poor quality; however, the following manual inspection of image quality revealed several images of poor quality. In the present study, staff were informed that the AI software would detect poor-quality images, but they were also trained to assess image quality themselves and retake suboptimal images before initiating the AI analysis [36]. However, we know from a connected study that a few of the staff did not assess image quality and relied on the AI software’s quality analysis [36], while others might have lacked the necessary competencies to evaluate quality themselves. It is to be mentioned that after an image is captured on the camera, it promptly displays the fundus image enlarged on the screen for two seconds before disappearing, requiring staff to manually reopen the image for a closer inspection. We suspect that a few of the staff did not take this extra step, resulting in image quality being judged within split seconds - likely to reduce the accuracy of these assessments. These findings highlight the need for more robust AI-based image quality assessment in primary care, while also emphasizing the importance of improving primary care staff competencies in capturing high-quality retinal images. Our findings raise important concerns and highlight a need for optimization prior to clinical implementation, as failure to consistently identify ungradable images may lead to suboptimal image quality being analyzed, thereby potentially affecting diagnostic safety in real-world screening.

The consequences of poor image quality became evident in the differences between Analysis 1 and Analysis 2, suggesting that image quality influences the AI system’s performance, although not in a uniform direction. In Analysis 2, sensitivity and NPV increased, indicating improved detection of true DR cases and fewer false negatives, whereas specificity and PPV decreased, reflecting more false positives. This likely reflects the small number of DR cases (*n* = 19, reduced to *n* = 15 in Analysis 2), where exclusion of even a few true positives can substantially affect performance estimates. Consequently, sensitivity and PPV are subject to considerable statistical uncertainty and should be interpreted with caution. The high NPV (>98%) is largely driven by the low disease prevalence rather than solely reflecting test performance.

This study provides a broader perspective on the differences between AI-assisted DRS in primary care and traditional ophthalmologist-led screening. Understanding not only what each setting enables, but also what it may fail to capture or identify, is essential for informed decision-making regarding the design and implementation of DRS programs. To support this, we included additional findings from the ophthalmologist medical report. It is to be mentioned that only one ophthalmologist assessed the additional findings, and the results should therefore be interpreted with caution. The reported findings were all conditions for which the risk is known to increase with age [41–43], and diabetes is further considered a risk for cataract[44] and glaucoma [45], whereas the association with AMD remains more uncertain [46]. The most common additional finding was non-specific optic nerve head and intraocular pressure observations, noted in 24 % of patients. These findings indicate risk factors the ophthalmologist considered relevant to document – not definitive Glaucoma diagnosis. The additional findings are possible because ophthalmologists perform more comprehensive examinations as part of standard DRS [38], and, with their expert knowledge, they can identify other ocular conditions. In contrast, in a primary care setting, the focus of AI-assisted DRS is limited to DR detection, and other ocular pathologies are therefore not identified. The additional findings reported in this study would therefore remain undetected in a primary care setup. However, the purpose of DRS in primary care is only to identify individuals with DR who require referral to an ophthalmologist, while patients still need to consult an ophthalmologist for other ocular conditions.

DRS protocols in primary care typically do not involve the use of mydriatics [21,24,25]. Many fundus camera models, including the one used in this study, are marketed as non-mydriatic. However, the absence of pharmacological pupil dilation can hinder image acquisition in certain cases or decrease image quality, for example in older patients, in those with cataract, or in suboptimal room lighting. In suboptimal lighting environments, as can occur in primary care settings [14], the pupil may constrict, making it more difficult to capture fundus images or ensure good image quality. To establish good image quality, some primary care studies have used mydriatics when adequate images could not be obtained without pupil dilation[20,29]. This solution could be considered in future primary care screenings.

One of the major advantages of implementing DRS in primary care is its potential to reach patients who are otherwise under-screened within the existing healthcare system. Danish national data reveals that 20% of eligible patients with T2D have never attending DRS and 30% have not attended within the past five years, indicating substantial gaps in screening coverage[47]. In our study, at least 17 patients (9.3%) had never previously undergone DRS, with the actual number likely higher given missing data for 26 additional individuals. The missing information is due to either the absence of prior screening or to a lack of data sharing between primary care and ophthalmology services. Due to this uncertainty, these were marked as missing. The high number of patients who have never attended DRS is noteworthy considering patients required to commit time and travel for follow-up DRS at the ophthalmologist practice and may be due to a close patient-provider relationship in primary care. Our findings support previous statements from both patients and healthcare professionals [8,11–14], suggesting that primary care might improve screening uptake and reach individuals who currently do not participate in DRS. Additionally, implementing DRS into primary care settings facilitates a more comprehensive chronic disease management, enabling individuals with T2D to receive coordinated care at a single point of contact.

As this was an early-stage study, no power calculation was conducted, and participants were limited to those aged 18–70 years. This may have influenced DR prevalence in our sample, as diabetes duration is a risk factors for DR [38,48]. The age restriction was applied due to the demanding study setup with multiple visits and to reduce some complexity in the DRS, as older patients could present more challenges in image acquisition due to other ocular conditions. In addition, the use of convenience sampling may introduce selection bias, and the restricted age range limits the generalizability of the findings to real-world screening populations. Results should therefore be interpreted considering these limitations. Future studies should include a broader age range and be based on formal sample size calculations. They should also report practical challenges, such as the proportion of patients for whom screening cannot be completed, to provide a more comprehensive understanding of DRS implementation.

Furthermore, an additional methodological limitation relates to the handling of discordant cases between human graders. Exclusion of these cases may introduce bias and potentially lead to an overestimation of diagnostic performance, as adjudication is typically used in diagnostic accuracy studies[33]. However, adjudication was not used due to practical constraints within this early-stage study setup, and exclusion was therefore applied. This approach may have influenced the reported diagnostic performance and should be considered when interpreting the results.

Ethnic variation can influence the prevalence of DR [49], and given that the Danish population is relatively homogeneous in terms of ethnicity, the generalizability of our findings to more diverse populations may be limited. Furthermore, as the study was conducted solely within Danish general practice, transferability to other countries may be limited due to differences in health care systems.

The AI software used in this study is marketed as a tool designed to assist lightly trained staff and is often used in optometric or ophthalmology settings. These professional groups typically operate in ideal screening setups and are likely to have more experience than the staff involved in this study. This represents a potential limitation in the study design, as the AI software was applied in a new setting based on the assumption that it could be directly transferred without the need for further adaptation. In addition, the system is based on detection of red lesions and does not assess other clinically relevant features of DR, such as exudates, cotton wool spots, or neovascularization. This differs from many contemporary deep learning-based systems, which are trained to detect a broader range of retinal features and may therefore achieve higher diagnostic performance.

## 5. Conclusion

AI-assisted DRS in primary care demonstrated potential to identify patients without DR, but with a considerable rate of false positives. Further validation in larger populations is required before this approach can be considered a safe rule-out strategy. Image quality was suboptimal in primary care and negatively affected diagnostic performance. Improving image acquisition through better staff training or more advanced AI-based quality assessment may enhance performance and reduce false positives. By integrating findings from ophthalmologists’ medical reports, this study highlighted key differences in screening outcomes between AI-assisted and traditional ophthalmologist-led screening.

Overall, these findings provide important groundwork for future research and may inform decisions regarding the implementation of AI-supported DRS in primary care.
